# Worsening Quality of Life in Young Adult, Highly Educated, and Married Female Patients with Vitiligo: A Hospital-Based Case Control Study in Taiwan

**DOI:** 10.3390/ijerph19116741

**Published:** 2022-05-31

**Authors:** Yuan-Ting Yang, Chung-Hao Hsu, Yu-Fen Wang, Yu-Jun Chang, Hui-Ju Yang, Jiunn-Liang Ko, Kuo-Chia Yang

**Affiliations:** 1 Institute of Medicine, Chung Shan Medical University, Taichung 40201, Taiwan; 156393@cch.org.tw; 2 Department of Pharmacy, Changhua Christian Hospital, Changhua 50006, Taiwan; 3 Department of Dermatology, Changhua Christian Hospital, Changhua 50006, Taiwan; 182277@cch.org.tw (C.-H.H.); 2544@cch.org.tw (K.-C.Y.); 4 Cancer Research Center, Department of Research, Changhua Christian Hospital, Changhua 50006, Taiwan; 82587@cch.org.tw; 5 Big Data Center, Epidemiology and Biostatistics Center, Changhua Christian Hospital, Changhua 50006, Taiwan; 83686@cch.org.tw; 6 Department of Post-Baccalaureate Medicine, College of Medicine, National Chung Hsing University, Taichung 40227, Taiwan; 7 Department of Medical Oncology and Chest Medicine, Chung Shan Medical University Hospital, Taichung 40201, Taiwan

**Keywords:** vitiligo, quality of life, Skindex-21, questionnaire, Taiwanese

## Abstract

Vitiligo is an acquired chronic depigmentation disorder that can have a negative impact on the quality of life (QoL). This is especially true for patients with non-white skin. Only few studies have investigated the QoL of Asian patients with vitiligo. We aimed to investigate the QoL in Taiwanese vitiligo patients and identify the factors that influence their QoL. The cross-sectional study recruited 100 vitiligo patients and 100 controls with general skin diseases in the Department of Dermatology of Changhua Christian Hospital. Data were obtained using a structured questionnaire for demographic information and modified Skindex-21 instruments. The QoL was not significantly different between vitiligo patients and controls. Among the vitiligo patients, adults exhibited deteriorated emotional levels and total QoL as compared with non-adults. Married females reported greater levels of emotional disturbance than the unmarried ones. A higher educational level and shorter history of disease were associated with greater emotional impacts. The patients with a generalized type of vitiligo suffered more in total QoL. After multivariate adjustment, the young adult patients aged 20–39 were associated with poorer total QoL. It is suggested that vitiligo patients who are aged between 20 and 39, are married females, are highly educated, have a shorter disease history, and suffer from the generalized type of this disease demonstrate more deterioration in their life quality compared with other vitiligo patients. Care providers should tailor the psychological counseling and treatment accordingly.

## 1. Introduction

Although several skin conditions are not life-threatening, they can have a significant impact on the patients’ quality of life (QoL) [1]. Vitiligo is an acquired chronic depigmentation disorder characterized by the development of well-demarcated white macules and patches on the skin due to the destruction of epidermal melanocytes. The depigmented areas may be localized or symmetrical and may coalesce into large, depigmented areas [2].

Many theories have been proposed as to the factors contributing to the pathogenesis of vitiligo, including genetic, autoimmune, neural, biochemical, oxidative stress, viral infection, and melanocyte detachment mechanisms; however, none of these factors can sufficiently and collectively explain the diverse phenotypes of this disease as well as its initiation and progression [3,4].

The contrast between the white patches of various sizes and shapes and the areas of normal skin is notable. A study in Germany showed that vitiligo has an impact on the individuals’ DLQI, social anxiety, social avoidance, helplessness, and anxious-depressive mood [5]. However, more than three-fourths of the cases recruited in that study had light skin type (types I, II, and III). In fact, several studies conducted in France, Saudi Arabia, Italy, the United States, Brazil, India, Iran, Tanzania, Tunisia, the UK, Korea, and Singapore showed that patients with vitiligo often experience stigmatization, social isolation, low self-esteem, and even deteriorating daily and functional activities [6]. For Asian countries, Chan et al. reported that Singaporean patients (*n* = 222) who were depressed or had thyroid disease were more likely to report poor quality of life [7]. Kim et al. found that patients with vitiligo (*n* = 133) were highly affected in the functional and emotional aspects of QOL and female patients showed worsened function scales [8]. Yang et al.’s study revealed that vitiligo, acne-induced erythema, and rosacea patients reported significantly worse QoL compared to lentigines or melasma patients as evaluated by DLQI, with vitiligo patients reporting the worst general health scores in the category of skin disorders with facial discoloration [9]. However, information on the QoL of vitiligo patients in Taiwan is still scarce; moreover, how the clinical factors, gender, educational status, age, and the extent of skin lesions are associated with different domains of QoL remains to be determined.

The psychiatric disorder burden in patients with vitiligo was underlined by the findings of a study in Taiwan, which revealed an increased risk of developing psychiatric diseases in both sexes, all age groups, and in all facility levels of care [10]. However, little is known about the QoL of Taiwanese vitiligo patients. Therefore, our study investigated the impact of vitiligo on the QoL of Taiwanese patients and aimed to understand the influence of this disease in Taiwan and to enable dermatologists to provide improved and more comprehensive medical care to patients.

## 2. Materials and Methods

The study was conducted at the Department of Dermatology of Changhua Christian Hospital; we randomly selected 100 outpatients with vitiligo as the experimental group (mean age: 34.6 ± 20.2 years; mean duration of disease: 7.4 ± 5.1 years; skin type: type III (*n* = 31) and type IV (*n* = 69) and 100 outpatients with other skin diseases as the control group (mean age: 28.1 ± 14.7 years; mean duration of disease: 5.4 ± 8.0 years). Vitiligo diagnosis was made clinically after taking patient history and a thorough body exam. Other skin diseases of the control group were defined as all skin diseases except vitiligo with any body part involvement, and the diseases in the recruited controls encompassed acne, alopecia areata, allergic contact dermatitis, atopic dermatitis, basal cell carcinoma, Darier’s disease, eczema, epidermal cyst, ganglion cyst, herpes simplex virus infection, herpes zoster, keloid, keratosis pilaris, lipoma, lichen simplex chronicus, nevus, pyogenic granuloma, post-inflammatory hyperpigmentation, systemic lupus erythematosus, syringoma, tinea unguium, tinea pedis, tinea versicolor, urticaria, and verruca.

All patients agreed to participate in the project and signed the informed consent form after the investigators explained the purposes of this study.

A total of 200 patients were asked to complete oral questionnaires that were administered by an interviewer. The interviewers were dermatologists with sufficient knowledge of both English and Chinese. The QoL of all the included patients was assessed based on the participants’ responses, which included both general personal data and detailed records of their family history, work patterns, and site of involvement. The classification of vitiligo type was defined as segmental, localized, or generalized, with the generalized type of vitiligo being classified as vitiligo that could not be classified as segmental or localized type. The involvement of vitiligo was evaluated by physicians and quantitatively measured as the body surface area (BSA). With regard to QoL assessment, we used the previously modified Skindex-21 [11], which comprises 21 questions in four dimensions: symptoms, emotions, life and social functions, and additional occupational functions (Supplemental Table S1 available via Zenodo at https://doi.org/10.5281/zenodo.6523036; accessed on 6 May 2022). Each question related to QoL required patients to rate the degree of irritation felt during the past month on a scale of 0 (never bothered) to 100 (always bothered). The higher the score is, the more impaired the QoL is. Adults were defined as being older than 20 years old, in accordance with the WHO statement. The individual educational level was divided into the higher group, consisting of college graduates or higher, and the lower group, consisting of high school graduates or below.

Patient data were recorded in Microsoft Excel 2016 sheets (Microsoft Corp, Seattle, WA, USA) and analyzed using IBM SPSS Statistics for Windows, Version 22.0 (IBM Corp., Armonk, NY, USA). *p*-values < 0.05 were considered statistically significant. In order to assess the quality of life of patients, we first explored the differences in various scale items between vitiligo patients and other skin condition patients. Then, for vitiligo patients, the total score of each scale was compared under different age, gender, education level, marital status, vitiligo involvement site, and vitiligo type categories. Student’s *t*-test was used for univariate analysis of continuous variables to determine whether there was a statistically significant difference between the vitiligo and control groups. Since vitiligo patients were regrouped according to different characteristics, they were all small samples; thus, the Mann–Whitney U test was used to test continuous variables between two independent samples and the chi-square test was used for categorical variables. Finally, a multiple linear regression analysis was used to control potential confounding factors to evaluate the impact of each related factor on the quality-of-life scales.

## 3. Results

### 3.1. No Significant Differences Were Observed in Emotions, Life and Social Functions, Occupational Function, and Total Life Quality in the Vitiligo and Control Groups

Patients with vitiligo reported equal levels of deterioration in their QoL and less disturbance by the manifested symptoms compared with patients with general skin diseases (Table 1). The vitiligo group had significantly fewer physical symptoms (itching, burning, hurting, and irritating) compared with the control group (9.4 vs. 21.9, *p* < 0.001), whereas no significant differences were observed in emotions, life and social functions, occupational function, and total life quality in both groups.

### 3.2. Emotional Disturbance and Impaired QoL Are More Common in Adults, Married Females, the Highly Educated, and Patients of Shorter Disease History

Within the vitiligo group, adults (median age: 42.0 years, 22.0–78.0) reported greater levels of deterioration in the emotional dimension and in the total QoL compared with non-adults (median age: 11.0 years, 2.0–18.0), whereas females reported equal levels of deterioration in all QoL dimensions as males (Table 2). Married patients in the vitiligo group reported greater emotional disturbances compared with non-married patients (44.3 vs. 27.1, *p* = 0.03). Among the female vitiligo patients, married participants felt more worried and concerned with their appearance compared to unmarried patients (80.0 vs. 50.0, *p* = 0.025, Table 3). Furthermore, the higher educational level group were revealed to be more affected, both emotionally and with respect to their life quality, compared with the lower educational level group. Finally, the shorter the disease history was, the more the emotional state of the vitiligo patients was affected (48.6 vs. 34.3, *p* = 0.048).

### 3.3. Patients with Generalized Type of Vitiligo Are Affected More Than Those with Nongeneralized by Emotion, Life and Social Functions, and QoL

With regard to the different types of vitiligo, patients with the generalized type of vitiligo had higher BSA involvement and were more significantly influenced by the disease compared with those suffering from the nongeneralized types with respect to their emotions, life and social functions, and total life quality (Table 4).

### 3.4. Head and Neck Involvement Is Associated with Poor QoL

The vitiligo patients with head and neck involvement demonstrated a higher level of disturbance in their life and social experiences than the vitiligo patients without head and neck involvement. In contrast, the vitiligo group with only arm involvement did not demonstrate greater disease-related impacts than those with vitiligo without arm involvement in all dimensions (Table 4).

### 3.5. Multivariate Adjustment Discloses That Being 20 to 39 Years Old Demonstrated a Significant Influence on the Total Vitiligo QoL

After multivariate adjustment, the generalized type of vitiligo, shorter disease history, and higher educational level were found to significantly impact the emotions scale. As for the functioning scale, the generalized type of vitiligo and involvement of the head and neck determined the influence on life and social activity after the adjustment. In the occupational scale, being 20 to 39 years old and with head and neck involvement caused the occupational deterioration. In the total scale, being 20 to 39 years old demonstrated a significant influence on the total vitiligo QoL based on the Skindex-21 evaluation (Table 5).

## 4. Discussion

Vitiligo is a chronic disease with an unpredictable natural course that significantly impacts the QoL of patients. However, it is inappropriate and inefficient to describe the severity of the disease by using only physical indicators, such as BSA or topographic distribution.

There are several instruments and methods that can evaluate QoL which are widely used in dermatological diseases as well as in studies on vitiligo, including both generic health-related QoL measures (i.e., Short-Form-36 and Short-Form-12, Nottingham Health Profile, Sickness Impact Profile, World Health Organization Quality of Life-100, and World Health Organization Quality of Life–BREF) and dermatology-specific health-related QoL measures (Dermatology Life Questionnaire Index (DLQI), Skindex-29, Skindex-16, Skindex-17, Dermatology Quality of Life Scales, and Dermatology-Specific Quality of Life) [12,13,14]. Selection of the appropriate instrument remains a trade-off between various psychometric properties and research objectives. In this study, we used the previously proposed Skindex-21 [11], which added five questions to Skindex-16 to elucidate the occupational dimension of the patients’ QoL.

In our study, vitiligo patients did not exhibit greater levels of QoL deterioration compared with patients with general skin diseases. This result is in agreement with the findings of Morrison et al.’s 2017 review study, which found no significant difference in the levels of life deterioration between vitiligo and acne and dermatitis patients [15].

A study conducted by Amer et al. in 2016 identified that married patients exhibited poorer QoL than unmarried patients [16]. Our study yielded similar results in that married patients with vitiligo exhibited greater emotional and appearance-related disturbances compared with unmarried patients with vitiligo. This intriguing finding contradicts the conventional concept that single people care more about their appearance. Furthermore, married patients with vitiligo were suggested to suffer elevated levels of marital stress, which could, in some cases, end in divorce under the perception of being “unclean” while posing a significantly negative influence on their sexual life and their relationships with other people [7,17,18,19]. We further compared the appearance and worry score between married and unmarried patients in separate male and female groups. Married and unmarried male patients demonstrated no significant differences in their appearance and worry scores, whereas married female patients experienced significantly greater impairments in appearance and worry compared with unmarried females. In addition, in females who developed vitiligo after marriage, their marital lives have been negatively influenced by vitiligo [20]. Dolatshahi et al.’s research also revealed that married females with vitiligo had a higher DLQI score compared with unmarried females [21].

In terms of the extent of the disease, a previous study conducted by Kim et al. demonstrated that the generalized distribution of vitiligo was considered to be a significant risk factor that could affect the patient’s sexual life [8]. Our results revealed that the emotions and daily functioning of patients with the generalized type of vitiligo were negatively affected. These findings indicate that the disease continues to have a significant impact on the patient’s life quality as the vitiligo areas enlarge and spread to other areas of the body.

Our study found greater levels of emotional stress in adults and in patients with a higher educational level. In the studies conducted by Mishra et al., the DLQI scores were significantly negatively correlated with education, and the authors suggested that a higher educational level can decrease the impact of vitiligo on QoL and can empower patients with vitiligo to embrace positive thinking in their lives [22]. However, Dolatshahi et al. found no significant correlation between the DLQI score and educational level of patients [21]. Bin Saif et al. found that vitiligo patients with a higher educational level experienced greater impairments in their QoL compared to those with a lower educational level. In fact, the authors speculated that highly educated patients and families have an increased awareness of the psychosocial implications of this disease, and thus, they are bound to experience significantly greater emotional and psychological stresses [23]. Moreover, the cultural differences among different populations seem to influence the general concept and anticipation of this disease. For instance, studies conducted in Western populations demonstrated greater impairment in groups with lower educational levels, a finding that opposes the results of studies conducted in Eastern populations [23]. Another study that demonstrated a greater burden on the QoL of vitiligo patients from a South Asian culture compared to those who were from an American culture validates the cultural difference in the impact of the disease [24]. In addition, dark-skinned vitiligo patients may show more color contrast compared with light-skinned vitiligo patients, a difference that caused the results obtained from Western and Eastern studies to vary. A vitiligo-specific burden tool taking into account skin phototype was further proposed by Ezzedine et al. to accommodate the difference between skin types [25]. Our study used Skindex-21 to evaluate vitiligo patients, and it was found that the disease has the greatest influence on patients’ emotions, especially in highly educated patients. Using similar measuring tools and similar skin types to our study, Bae et al. used the Skindex-29 and found that higher educational level was independently associated with impaired emotional QoL in a Korean population [26].

The vitiligo patients did not demonstrate greater levels of deterioration compared with the control group in our study; however, vitiligo patients aged 20–39 years and patients with head and neck involvement demonstrated increased occupational difficulties after multivariate adjustment analysis. The majority of previous studies conducted on this topic indicated a significant reduction in work and study over item 7 of the DLQI; however, this item was still the lowest compared with the other items [27,28]. Wong and Baba evaluated the difference between workers and non-workers and found that working patients had significantly higher DLQI scores than those who had already retired [29]. Our study used five additional detailed questions to Skindex-21 to evaluate the impact of vitiligo on job aspects and thus provides an alternative method to register occupational deterioration.

Young adults are suggested to have more occupational and social activities; thus, the vitiligo disease might influence their interactions and self-esteem more. Mashayekhi et al. and Radtke et al. found that the highest QoL impairment was observed during the third decade of life [28,30], while some studies found no influence of age [18,22]. Our study demonstrated that the vitiligo patients aged between 20 and 39 years experienced more disturbance in the total and occupational life quality.

Dermatological and psychiatric diseases can be closely related. Stress causes dermatological diseases and increases the severity of skin symptoms, whereas dermatological diseases can have a distinct psychological impact and cause certain psychiatric symptoms. Furthermore, the immune system and psychiatric disorders are also known to be inter-related [31,32,33]. For example, depression has been reported to play a major role in various stages of inflammation by affecting the number and function of cells in the immune system and by altering the release of pro-inflammatory cytokines [31]. Physiological factors causing and mediating emotional stress are considered to induce the formation of or aggravate various dermatological diseases [34,35]. In a previous study conducted in Taiwan, a detailed psychiatric evaluation of patients with vitiligo indicated that 90% of the patients had at least one psychiatric disease. In addition, most patients reported an active psychostressor at the onset of the lesions. These findings emphasize the importance of psychiatric evaluation and management of vitiligo [10].

Our study has some limitations. Firstly, the vitiligo group was older than the control group, which might have caused biased responses pertaining to the QoL, expectancy, work, and social relationships. Secondly, although the control group in our study may be considered to reflect the patients we encounter in the outpatient medical setting with all the skin diseases in normal distribution, the small sample size and heterogeneity of the control group might hamper the comparison between the vitiligo group and controls, and studies with a larger sample size are needed to clarify the difference between the vitiligo group and controls and to verify the differences within the vitiligo group.

## 5. Conclusions

Vitiligo patients who were aged between 20 and 39, were married females, were highly educated, had a shorter disease history, and suffered from the generalized type of this disease demonstrated more deterioration in their life quality compared with other vitiligo patients. Our findings emphasize that patients with vitiligo are little affected by the manifestation of vitiligo-related symptoms; instead, they experience distinct psychosocial complications. Moreover, the aspects of this disease that were found to influence the QoL in vitiligo patients may not be the same among different ethnic groups and cultures, and how these factors result in the deteriorated quality of life needs more investigation. Therefore, to improve the QoL and subsequent treatment outcomes, physicians should consider the psychological effects of this disease on patients, and a complete treatment plan for vitiligo patients should focus more on psychological counseling than disease treatment.

## Figures and Tables

**Table 1:** The modified Skindex-21 questionnaire survey results for patients with vitiligo.

	Vitiligo (*n* = 100)	Control (*n* = 100)	*p*-Value ^a^
Age	34.6 ± 20.2	28.1 ± 14.7	0.01
Body surface area involved (%)	5.1 ± 7.8 (*n* = 97)		
Duration of disease	5.1 ± 7.4	5.4 ± 8.0	0.771
Symptoms scale	9.4 ± 16.3	21.9 ± 23.6	<0.001
Itching	11.9 ± 20.5	36.2 ± 34.1	<0.001
Burning/stinging	5.9 ± 17.2	14.0 ± 25.6	0.009
Hurting	3.7 ± 15.5	10.9 ± 22.9	0.01
Irritated	16.0 ± 27.6	26.7 ± 35.4	0.018
Emotions scale	43.0 ± 29.1	40.2 ± 28.9	0.489
Persistence/recurrence	47.6 ± 36.0	42.5 ± 38.8	0.338
Worry	59.7 ± 36.2	55.9 ± 37.3	0.465
Appearance	59.0 ± 37.6	52.5 ± 38.4	0.224
Frustration	39.2 ± 36.4	36.0 ± 34.6	0.531
Embarrassment	40.7 ± 37.8	38.1 ± 32.7	0.616
Annoyed	25.1 ± 34.2	28.9 ± 33.3	0.425
Depressed	29.9 ± 34.7	27.3 ± 32.6	0.578
Functioning scale	16.8 ± 25.4	16.6 ± 21.1	0.948
Interactions with others	24.1 ± 34.5	20.3 ± 28.3	0.385
Desire to be with people	15.5 ± 28.2	10.9 ± 22.1	0.196
Show affection	13.8 ± 28.0	8.1 ± 19.3	0.095
Daily activities	14.5 ± 26.6	23.0 ± 30.2	0.037
Work/What you enjoy	16.3 ± 28.5	21.0 ± 29.2	0.249
Occupational scale	6.0 ± 16.3	5.0 ± 13.4	0.649
May need to leave job	6.2 ± 18.8	6.6 ± 19.9	0.884
Fear of being fired	5.7 ± 17.9	4.7 ± 17.4	0.689
Financial future	5.7 ± 19.1	4.1 ± 15.1	0.512
Interactions with co-workers	8.5 ± 21.2	4.6 ± 14.6	0.132
Difficulty using hands	3.8 ± 13.6	5.1 ± 15.6	0.531
Total scale	18.8 ± 16.6	20.9 ± 17.4	0.376

The data are presented as mean ± standard deviation. ^a^ *p*-value by Student’s *t*-test.

**Table 2:** Correlation of QoL in patients with vitiligo.

	QoL, Median (Range)				
	Symptom Scale	Emotion Scale	Functioning Scale	Occupational Scale	Total Scale
**Age, years**					
<20 (*n* = 27)	0.0 (0.0–25.0)	18.6 (0.0–99.7)	0.0 (0.0–100.0)	0.0 (0.0–60.0)	5.3 (0.0–71.2)
20–39 (*n* = 73)	2.5 (0.0–82.5)	45.7 (1.4–100.0)	4.0 (0.0–100.0)	0.0 (0.0–80.0)	19.3 (1.1–79.3)
*p*-value ^a^	0.076	0.006	0.091	0.059	0.001
**Gender**					
Male (*n* = 45)	2.5 (0.0–82.5)	34.3 (0.0–91.4)	0.6 (0.0–92.0)	0.0 (0.0–80.0)	13.5 (0.0–79.3)
Female (*n* = 55)	0.0 (0.0–57.5)	44.3 (0.0–100.0)	2.0 (0.0–100.0)	0.0 (0.0–80.0)	16.4 (0.4–71.2)
*p*-value ^a^	0.328	0.199	0.822	0.269	0.563
**Marital status**					
Married (n = 59)	2.5 (0.0–57.5)	44.3 (1.4–100.0)	2.0 (0.0–100.0)	0.0 (0.0–80.0)	18.0 (1.1–58.4)
Unmarried (n = 41)	2.5 (0.0–82.5)	27.1 (0.0–99.7)	0.0 (0.0–100.0)	0.0 (0.0–80.0)	9.2 (0.0–79.3)
*p*-value ^a^	0.788	0.030	0.684	0.534	0.093
**Education**					
High school level or below (*n* = 45)	0.0 (0.0–75.0)	27.1 (0.0–100.0)	0.0 (0.0–90.0)	0.0 (0.0–80.0)	7.4 (0.0–46.4)
College level or higher (*n* = 55)	5.0 (0.0–82.5)	51.4 (2.9–100.0)	6.0 (0.0–100.0)	0.0 (0.0–80.0)	20.1 (1.1–79.3)
*p*-value ^a^	0.005	0.001	0.066	0.061	0.001
**Vitiligo history, years**					
<2 (*n* = 45)	2.5 (0.0–75.0)	48.6 (0.0–100.0)	2.0 (0.0–100.0)	0.0 (0.0–80.0)	20.0 (0.4–71.2)
≥2 (*n* = 55)	0.0 (0.0–82.5)	34.3 (0.0–95.7)	0.0 (0.0–92.0)	0.0 (0.0–80.0)	10.6 (0.0–79.3)
*p*-value ^a^	0.642	0.048	0.903	0.637	0.173

^a^*p*-value by Mann–Whitney U Test.

**Table 3:** Correlation of marital status with QoL of female vitiligo patients.

	Marital Status		
Scale, Median (Range)	Married Female (*n* = 36)	Unmarried Female (*n* = 19)	*p*-Value ^a^
Symptoms scale	0.0 (0.0–57.5)	50.0 (0.0–25.0)	0.939
Emotions scale	59.3 (1.4–100.0)	24.3 (0.0–99.7)	0.056
Worry	80.0 (0.0–100.0)	50.0 (0.0–100.0)	0.014
Appearance	80.0 (0.0–100.0)	50.0 (0.0–100.0)	0.025
Functioning scale	10.0 (0.0–100.0)	0.0 (0.0–100.0)	0.585
Occupational scale	0.0 (0.0–80.0)	0.0 (0.0–60.0)	0.432
Total scale	20.8 (1.1–58.4)	11.4 (0.4–71.2)	0.100

^a^*p*-value by Mann–Whitney U Test.

**Table 4:** Impact of vitiligo involvement on QoL.

	Vitiligo Type			Vitiligo with Head and Neck Involvement		
Scale, Median (Range)	Nongeneralized (*n* = 69)	Generalized (*n* = 31)	*p*-Value ^a^	No (*n* = 21)	Yes (*n* = 79)	*p*-Value ^a^
BSA (%)	2.0 (0.3–15.0)	5.0 (1.0–50.0)	<0.001			
Symptoms scale	2.5 (0.0–82.5)	0.0 (0.0–62.5)	0.997	2.5 (0.0–75.0)	2.5 (0.0–82.5)	0.620
Emotions scale	34.3 (0.0–99.7)	52.9 (7.1–100.0)	0.021	35.7 (0.0–97.1)	42.9 (0.0–100.0)	0.725
Functioning scale	0.0 (0.0–100.0)	18.0 (0.0–100.0)	0.004	0.0 (0.0–40.0)	4.0 (0.0–100.0)	0.038
Occupational scale	0.0 (0.0–80.0)	0.0 (0.0–44.0)	0.305	0.0 (0.0–20.0)	0.0 (0.0–80.0)	0.182
Total scale	11.7 (0.0–79.3)	22.8 (1.8–58.4)	0.015	11.4 (0.0–42.5)	13.9 (0.0–79.3)	0.400

^a^*p*-value by Mann–Whitney U Test.

**Table 5:** Results of generalized linear model on each scale in patients with vitiligo.

Scale	Predictor		Estimate	SE	95% CI			*p*-Value
Emotions	(Intercept)		36.50	6.12	24.51	-	48.50	<0.001
	Vitiligo type	Generalized	14.66	5.81	3.27	-	26.04	0.012
		Nongeneralized	0.00					
	Vitiligo history	>2 years	−13.13	5.39	−23.69	-	−2.57	0.015
		<2 years	0.00					
	Education	College or above	19.44	6.55	6.61	-	32.27	0.003
		Senior high school	16.23	7.44	1.64	-	30.82	0.029
		Junior high school	6.68	8.63	−10.24	-	23.60	0.439
		Elementary school or below	0.00					
	Family history	Yes	−12.04	6.53	−24.84	-	0.76	0.065
		No	0.00					
Functioning	(Intercept)		2.44	5.40	−8.15	-	13.03	0.651
	Vitiligo type	Generalized	14.11	5.15	4.02	-	24.21	0.006
		Nongeneralized	0.00					
	Head and neck involvement	Yes	12.69	5.85	1.23	-	24.16	0.030
		No	0.00					
Occupational	(Intercept)		−3.08	4.06	−11.05	-	4.88	0.448
	Age	60–79	−3.02	5.52	−13.85	-	7.80	0.584
		40–59	−0.06	4.11	−8.11	-	8.00	0.989
		20–39	10.11	3.92	2.43	-	17.79	0.010
		<20	0.00					
	Head and neck involvement	Yes	7.56	3.81	0.09	-	15.03	0.047
		No	0.00					
Total	(Intercept)		11.83	3.00	5.96	-	17.71	<0.001
	Age	60–79	−2.61	5.65	−13.69	-	8.47	0.645
		40–59	5.42	4.43	−3.26	-	14.11	0.221
		20–39	10.87	4.00	3.03	-	18.72	0.007
		<20	0.00					
	Vitiligo type	Generalized	6.58	3.59	−0.46	-	13.61	0.067
		Nongeneralized	0.00					

## Data Availability

The datasets generated and analyzed during the current study are not publicly available due to maintenance of confidentiality and privacy requirements consistent with the Institutional Review Board approval.

## Supplementary Materials

The following supporting information can be downloaded via Zenodo at: https://doi.org/10.5281/zenodo.6523036 (accessed on 6 May 2022), Table S1: Modified Skindex-21 questionnaire for quality of life.
